# A Whole-Cell Biosensor for the Detection of Gold

**DOI:** 10.1371/journal.pone.0069292

**Published:** 2013-08-09

**Authors:** Carla M. Zammit, Davide Quaranta, Shane Gibson, Anita J. Zaitouna, Christine Ta, Joël Brugger, Rebecca Y. Lai, Gregor Grass, Frank Reith

**Affiliations:** 1 The University of Adelaide, School of Earth and Environmental Sciences, Centre of Tectonics, Resources and Exploration (TRaX), Adelaide, South Australia, Australia; 2 The Commonwealth Scientific and Industrial Research Organisation (CSIRO) Land and Water, Environmental Biogeochemistry, PMB2, Glen Osmond, South Australia, Australia; 3 The University of Nebraska-Lincoln, School of Biological Sciences, Lincoln, Nebraska, United States of America; 4 The University of Nebraska-Lincoln, Department of Chemistry, Lincoln, Nebraska, United States of America; 5 Flinders University, School of Chemical and Physical Sciences, Adelaide, South Australia, Australia; 6 Mineralogy, South Australian Museum, Adelaide, South Australia, Australia; 7 Bundeswehr Institute of Microbiology, Munich, Germany; RMIT University, Australia

## Abstract

Geochemical exploration for gold (Au) is becoming increasingly important to the mining industry. Current processes for Au analyses require sampling materials to be taken from often remote localities. Samples are then transported to a laboratory equipped with suitable analytical facilities, such as Inductively Coupled Plasma-Mass Spectrometry (ICP-MS) or Instrumental Neutron Activation Analysis (INAA). Determining the concentration of Au in samples may take several weeks, leading to long delays in exploration campaigns. Hence, a method for the on-site analysis of Au, such as a biosensor, will greatly benefit the exploration industry. The *golTSB* genes from *Salmonella enterica* serovar typhimurium are selectively induced by Au(I/III)-complexes. In the present study, the *golTSB* operon with a reporter gene, *lacZ*, was introduced into *Escherichia coli*. The induction of *golTSB*::*lacZ* with Au(I/III)-complexes was tested using a colorimetric β-galactosidase and an electrochemical assay. Measurements of the β-galactosidase activity for concentrations of both Au(I)- and Au(III)-complexes ranging from 0.1 to 5 µM (equivalent to 20 to 1000 ng g^−1^ or parts-per-billion (ppb)) were accurately quantified. When testing the ability of the biosensor to detect Au(I/III)-complexes_(aq)_ in the presence of other metal ions (Ag(I), Cu(II), Fe(III), Ni(II), Co(II), Zn, As(III), Pb(II), Sb(III) or Bi(III)), cross-reactivity was observed, *i.e.* the amount of Au measured was either under- or over-estimated. To assess if the biosensor would work with natural samples, soils with different physiochemical properties were spiked with Au-complexes. Subsequently, a selective extraction using 1 M thiosulfate was applied to extract the Au. The results showed that Au could be measured in these extracts with the same accuracy as ICP-MS (P<0.05). This demonstrates that by combining selective extraction with the biosensor system the concentration of Au can be accurately measured, down to a quantification limit of 20 ppb (0.1 µM) and a detection limit of 2 ppb (0.01 µM).

## Introduction

In recent years the market price of Au has steadily increased and currently stands at approximately USD $1,600 per ounce (2013). This price rise has been driven by the growing demand for Au: for use in jewellery, particularly in middle-eastern and east Asian countries; for components in modern technologies; and, as a form of investment and security for governments and the financial sector [1]. The price and demand of Au may be rising, but the supply of Au is stagnating and exploration for new deposits is becoming less successful [2].

In spite of the progress achieved using geochemical and geophysical techniques, exploration for new Au deposits is technically challenging [3]. In recent years the discovery of world-class Au deposits has been sporadic and rare. The main reason is that outcropping deposits and those with obvious geophysical and geochemical signatures have already been discovered [4]. Hence, Au exploration in many countries is journeying into landscapes where thick layers of *in situ* or transported weathered materials (regolith) cover deeply buried mineralization [2], [5]. In these areas, weathering of the underlying deposits and prolonged dispersion of metals has left geochemical haloes of Au and its pathfinder elements such as Ag, As, Bi, Mo, Pb, Se and W [6] in overlying soils and weathered material (such as calcrete or ferricrete) [7], [8]. Therefore, to successfully explore for Au in these uncharted terrains, new techniques are required to increase the success of exploration campaigns.

Geophysical methods are commonly used for the initial identification of areas with prospective Au mineralization [4]. Subsequent geochemical sampling targets particular types of surface materials, *e.g.*, soils, calcrete or ferricrete [7], [9]. These samples are transported to a laboratory, extracted using total selective extraction techniques and analyzed for Au and its ‘pathfinder’ elements [10], [11]. Particular selective extraction techniques are now commonly used as they allow for the extraction of Au that is associated within operationally-defined phases, *e.g.* organics, iron oxides, clays or carbonates, and soil or regolith materials [9], [11], [12], [13], [14]. To achieve this, the use of solutions containing lixiviants, such as, thiosulfate, hydroxylamine-hydrochloride or sodium pyrophosphate is common [9]. Techniques such as Inductively Coupled Plasma-Mass Spectrometry (ICP-MS) or Instrumental Neutron Activation Analysis (INAA) are then used to detect and quantify Au in extraction solutions or solid samples, respectively. The results from these analyses are reported back to exploration teams and decisions in regards to further investigation are evaluated. The entire process can take up to several weeks and requires complex analytical instrumentation used in dedicated laboratories. Hence, a reliable on-site test for Au will be of great benefit, as it will provide an on-the-spot assessment of Au in the area, allowing geologists to ‘hone-in’ on areas of interest.

The development of biosensing technologies for mineral exploration holds value in the speed, portability and potentially high selectivity and sensitivity of the assay [15], [16]. Additionally, biosensing devices may aid in mineral processing, providing ‘in-line’ analysis of ores and process waters, and assisting in the tailoring of the processing method to maximize Au extraction and minimize chemical consumption. To date, research into biosensors has focused around the detection and monitoring of heavy metal contamination, blood glucose levels, pathogens, food toxins, and illicit drugs [16]. Conversely, little research has been conducted on the use of biosensors for the minerals industry [17]. A biosensor consists of a biological element that is affected by an external stimulus: this effect is then manipulated into a measurable signal, which is used to elucidate qualitative or quantitative information about the initial stimuli [16]. For the development of a Au biosensor, biological elements that respond to Au are required.

To develop a commercially applicable Au biosensor for geochemical exploration campaigns a number of challenges have to be overcome. One of these challenges comes from the fact that the concentration and speciation of Au (*i.e.*, oxidation state, complexing ligand and stability of aqueous complexes) determines its toxicity to cells [18], [19]. In contrast to other heavy metals, Au does not form free ions in aqueous solution at surface conditions, but occurs in solution only as aurous (I) or auric (III) complexes [20]. Based on thermodynamic calculations and natural abundances of possible ligands, complexes with chloride, thiosulfate, ammonium and cyanide appear to be the most important Au complexes under surface conditions (*e.g.*, [6]). Hence, the speciation of Au, and not only its concentration, determines its toxicity, and consequently the genetic and biochemical responses of biosensors [19].

Another issue may lie in an unspecific microbial responses to toxic metals, highlighting one of the major challenges for the development of useful biosensors. Recent research has shown that the metallophillic bacterium *Cupriavidus metallidurans*, which forms biofilms on Au grains, rapidly accumulates Au-complexes from solution into the cell [18], [21], [22], [23]. This is the result of Au(I/III)-regulated gene expression, leading to energy-dependent reductive precipitation of toxic Au-complexes [18]. However, most of the defence mechanisms utilized by bacteria, such as *C. metallidurans*, are nonspecific responses to ranges of toxic metals, such as Cu(II) or Ag(I), rather than just Au(I/III) [18], [19], [24].

In contrast, *Salmonella enterica* serovar typhimurium strain LT2 contains the *golTSB* regulon, which holds potential as the biological basis of a Au biosensor [25]. The *golTSB* operon works to detoxify the cell from harmful Au(I)-and Au(III)-complexes in the following way: GolS regulates the transcription of the other two genes in the operon; GolT is a transmembrane efflux ATPase; and GolB acts as a metallochaperone. Checa *et al*. (2007) [25] found that expression of *golB* increased in the presence of Au(III)-complexes, but not in the presence of other metal ions such as Cu(II), Ag(I), Zn(II), Cd(II), Hg(II), Fe(II), Co(II), Ni(II), Pb(II), making the *golTSB* regulon an ideal chassis for the development of a Au biosensor for geochemical exploration. A biosensor system, developed by Checa *et al.* (2011), was capable of detecting but not quantifying Au: quantification is crucially important for geochemical exploration [26]. In addition, implementation of a biosensor on field samples is often met with little success due to the complex composition of environmental samples. Thus, an extraction method needs to be applied that solubilizes Au from the solid phases resulting in a Au complex that is able to induce the biosensor.

The aims of this study were to: (a) heterologously express the *golTSB* genes from *S. enterica* serovar typhimurium in *E. coli* and fuse them to a promoter-less *lacZ* reporter gene, under the control of the GolS regulator; (b) investigate to what extent this construct can be used as a sensitive biosensor in determining concentrations of Au(I)- and Au(III)-complexes; (c) test the sensors selectivity to detect Au(I) and Au(III) when in combination with other mobile metal species relevant for mineral exploration, such as Cu(II), Ag(I), Fe(III), Ni(II), Co(II), Zn(II), As(III), Pb(II), Sb(III) and Bi(III); (d) assess the speciation of Au-complexes in induction solutions; (e) test the biosensor system in combination with a selective extraction technique applied to field samples.

## Materials and Methods

### Strains and growth conditions

All cultures were grown at 37°C (or 30°C for *E. coli* harbouring the plasmid pINT-ts) on Luria-Bertani (LB) broth with shaking at 120 revolutions per minute (rpm) or LB Agar (Difco BD Bioscience). Selection was established with 35 µg mL^−1^ kanamycin (Km) or with 100 µg mL^−1^ ampicillin (Amp). *E. coli XL-1* DH5α and EC100D pir-116 were used to propagate plasmids. Wild-type *E. coli* strain K12 (W3110) was used to construct the biosensor.

### Plasmid construction

PCR fragments for cloning were constructed using primers GolTopF (5′- AAACT GCAGC GGCGA TGACG CCGGC TATCA -3′) and GolBstopR (5′- AAAGA ATTCT ACCTC TCGCG CGGCG GGAAA C -3′). When cut with PstI/EcoRI the resulting fragment contains the entire *gol* operon, ranging from 200 bp upstream of the *golT* start codon to the stop codon of *golB*. All restriction enzymes were from Promega or New England Biolabs. Plasmid pAH125-golReg was constructed following the general cloning procedure as outlined in Haldimann and Wanner (2001) [27]. The plasmid was then integrated into the *E. coli* W3110 genome, using the helper plasmid pINT-ts, providing the lambda phage integration functions, as described [27] (Fig. S1).

### Induction of the biosensor with metal ions

All metal stock solutions were freshly prepared by dissolution in deionized water, ammonia (Fluka) or *aqua regia* (3 parts HCl: 1 part HNO_3_). The solutions were then diluted to a working concentration and the pH adjusted to 7.0 prior to use. A single colony was picked from LB agar plates and grown in minimal salts medium (MSM) [28] with 0.2 wt.% glycerol or 0.2 wt.% glucose. Cells were allowed to reach log phase (approximately 2 h) then 3 mL of culture was added to 16×100 mm tubes (VWR, fitted with a KIMAKAP cap). To this 0, 0.05, 0.1, 0.5, 1.0, 2.0, 3.0, 4.0, 5.0, 6.5, 8.0 and 10.0 µM of Au(I) (as Au(I)-thiosulfate, Sigma-Aldrich), 0, 0.05, 0.1, 0.5, 1.0, 2.0, 3.0, 4.0, 5.0, 6.5, 8.0 and 10.0 µM of Au(III) (as AuHCl_4._3H_2_O, Sigma-Aldrich) were added. For cross reactivity testing 5.0 µM of AgCl, CuCl_2_ (Sigma-Aldrich), FeCl_3_ (Fisher Chemicals), NiCl_2_ (Sigma-Aldrich), CoCl_2_ (Sigma-Aldrich), ZnCl_2_ (Sigma-Aldrich), AsCl_3_ (Acros Organics), PbCl_2_ (Acros Organics), SbCl_3_ (Strem Chemicals) or BiCl_3_ (Acros Organics) were added to cultures with 0.5 µM of Au(I) or Au(III). Cultures were incubated for 16 h with shaking at 120 rpm and 37°C. Cultures were then placed on ice for 20 min. All cultures were grown in triplicate for biosensor assays.

### Selective extraction

To test the biosensor system with environmental samples, selective extractions were applied to duplicates of Au-spiked soils. Soils from four physicochemical distinct Australian sites not containing natural Au were used (Table S1): Nullarbor (high soil-pH, high clay content, carbonate-rich), Frankland (low soil-pH, low clay content), Ora Banda (OB; low soil-pH, high clay content) and Tomakin (low soil-ph, organic-rich). Soils (10 g) were weighed and transferred to 50 mL centrifuge tubes, spiked with 10 mL of 0.1 µM Au(III)-chloride_(aq)_ solution and shaken at room temperature for 10 days. Soils were dried overnight in a 60°C oven. Subsequently, soils were subjected to an overnight extraction using 10 mL of 1 M sodium-thiosulfate solution on an overhead shaker, in the dark. Soils were centrifuged at 3000 rpm at 4°C for 30 min and the supernatant was decanted. Finally, the supernatant was syringe filtered with a sterile 0.22 µM filter. ICP-MS (Agilent 7500ce, Agilent Technologies; CSIRO Land and Water, Adelaide, Australia) was used to determine the concentration of Au in the extraction solution. The extraction solutions were used to induce the biosensor. Based on the ICP-MS results, solutions were diluted to 0.5 µM of Au (a dilution factor of approx. 200) using MSM. Triplicates of each of the extractions were subsequently analyzed using the biosensor.

### β-galactosidase assay

Initial testing of mutants and their responses to Au(I/III)-complexes was carried out using a standard β-galactosidase assay [29] with minor changes. In short, the following procedure was carried out in a 30°C temperature controlled room. To 10 µL of culture the following components were added, in the order listed: 1 mL of Buffer Z (10 mM KCl, 1 mM MgSO_4_, 2.7 mL L^−1^ mercaptoethanol, freshly prepared in sodium phosphate buffer); 100 µL of chloroform; and 50 µL of 0.1% SDS. The cells were then mixed by vortex and 200 µL of 4 mg mL^−1^ ortho-nitrophenyl-β-galactoside (ONPG) was added. The specific β-galactosidase activity was expressed as Miller Units (MU) [30].

### Electrochemical assaying

Electrochemical detection of the presence of trace metal complexes is also possible through the use of the same reporter enzyme, β-galactosidase. It takes the substrate p-aminophenyl-β-D-galactopyranoside (PAPG), which is electrochemically inactive, but when converted by β-galactosidase to p-aminophenyl (PAP), is electrochemically active as an electron donor. The electrode, at a constant potential of 220 mV vs. Ag/AgCl, can then permanently oxidize enzyme-generated PAP to 4-iminocyclohexa-2,5-dienone. This allows for real time detection of PAP. The substrate PAPG was not added to the electrode until 15 s into the run to reduce the non-faradaic signal. A single scan was taken in 180 s and the change in the current over time was measured.

To conduct these measurements using the electrochemical system, the clone of the biosensor that resulted in the greatest sensitivity for Au(I/III)-complex detection using the β-galactosidase assay was chosen for further testing. In addition to testing the sensitivity of the system to Au(I/III)-complexes alone, cross reactivity of Cu(II), Ag(I), Fe(III), Ni(II), Co(II), Zn(II), As(III), Pb(II), Sb(III), Bi(III) and Au(I) or Au(III) as well as Au extracted selectively from spiked field samples was tested using the electrochemical assays.

All electrochemical assays were conducted using the following procedure: after 20 min on ice, 1 mL of cell culture was transferred to a 1.5 mL centrifuge tube and centrifuged at maximum speed for 60 s. The supernatant was removed and cells were re-suspended in 1 mL of phosphate buffer (PB, pH 7.4), 50 µL 10% SDS and 100 µL chloroform (in that order) then mixed by vortex for 30 s. This was centrifuged at maximum speed for 60 sec and 900 µL of this was added to the electrochemical chamber followed by 100 µL of 4 mg mL^−1^ electrogenic substrate PAPG. The reagents potassium ferricyanide and potassium ferrocyanide were used as received (Sigma-Aldrich). All other chemicals were of analytical grade and made with deionized water purified through a Milli-Q system (DI; 18.2 MΩ•cm, Millipore).

Electrochemical measurements were performed at room temperature using a CHI 1040A or 600D Electrochemical workstation with three electrodes (CH Instruments). The working electrode was a glassy carbon electrode (GCE) with a geometric area of 007.068 mm^2^. The counter electrode was a platinum (Pt) wire. The pseudo-reference electrode was a Ag wire that was calibrated against a Ag/AgCl (3 M KCl) reference electrode using ferricyanide/ferrocyanide as the redox standard (2.5 mM ferricyanide, 2.5 mM ferrocyanide, 8.02 mM Na_2_HPO_4_, 1.98 mM NaH_2_PO_4_, 1 M NaCl, pH 7.4).

The GCE was polished using 1 µm aqueous diamond slurry (Buehler) to a mirror finish. The electrode was then rinsed with DI water and sonicated for 30 s to remove bound particulates. Prior to the chronoamperometric experiments, the Ag pseudo-reference electrode was first calibrated using the ferricyanide/ferrocyanide redox couple. To oxidize the enzymatic product, p-aminophenol, to p-iminoquinone, an oxidizing potential of +220 mV (vs. Ag/AgCl) was applied to the GCE. Pure β-galactosidase and the substrate, PAPG, were added to the buffer solution approximately 15 s after the potential was applied. Measurements were conducted in triplicates for all enzyme concentrations used in this study and the slopes of the chronoamperograms (*i.e.*, current-time plots) were used to generate a calibration plot for β-galactosidase enzymatic activity. The same procedure was used to measure the activity of β-galactosidase in cell lysates of *E. coli* grown with the various metals and selective extractions.

### Geochemical modeling

To establish the initial composition of aqueous Au species in the MSM is important, because Au speciation influences cellular responses, and it cannot be assumed that the addition of a solid Au compound, *e.g.*, sodium-tetrachloroaurate(III)-hydrate or sodium-gold(I)-thiosulfate hydrate to MSM will result in the formation of Au(III)-chloride(aq) and or Au(I)-thiosulfate(aq) complexes. Hence, a chemical model for the speciation of sodium-tetrachloroaurate(III)-hydrate and sodium-gold(I)-thiosulfate hydrate in MSM was calculated. Calculations were conducted using Geochemist's Workbench (GWB; [31]). Thermodynamic properties were taken from the Lawrence Livermore National Laboratory database (version R9), and properties for gold complexes from Usher *et al*. [32].

### Data analysis and statistics

Microsoft Excel was used to graph the data, and the inbuilt function was used to determine the lines of best fit, their subsequent equations and R^2^ values. The one tailed t-test was used to calculate the difference between means when investigating the differences between measurements taken with and without additional metals, P<0.05 and P<0.10 was used as significance cut off values, as reported in results. Analysis of variance (ANOVA) was used to calculate differences between gold measured using ICP-MS and the biosensor.

## Results

### Transgenic biosensor induction by Au(I)- and Au(III)-complexes

**Figure 1:**
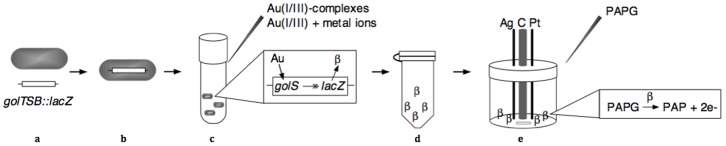
Schematic of methodology employed to build the Au biosensor. (a) The *golTSB* regulon is regulated by Au ions in *Salmonella enterica* serovar *typhimurium*. A synthetic *golTSB* regulon was made by fusing a promoter-less *lacZ* reporter gene downstream of the *golB* open reading frame as a transcriptional fusion. (b) This *golTSB*::*lacZ* transcriptional fusion was introduced as a single copy into the chromosome of *E. coli*. (c) A single clone was taken for further studies and incubated overnight, used to inoculate new media, then metals (various concentrations of Au(I) or Au(III) or cross reactivity testing with Cu(II), Ag(I), Fe(III), Ni(II), Co(II), Zn(II), As(III), Pb(II), Sb(III) and Bi(III) and Au(I) or Au(III)) were added and this was incubated for 16 h. (d) Cells were permebabilized for access to the β-galactosidase produced by the *lacZ* gene in the presence of Au. (e) The permeabilized cells were transferred to the electrochemical cell, p-aminophenyl-β-D-galactopyranoside (PAPG) was added. The β-galactosidase cleaves PAPG to p-aminophenol (PAP), the PAP is then oxidized by the carbon electrode vs. Ag/AgCl giving off electrons (e-) and the slope of the current-time plots were used to determine β-galactosidase enzymatic activity.

**Figure 2:**
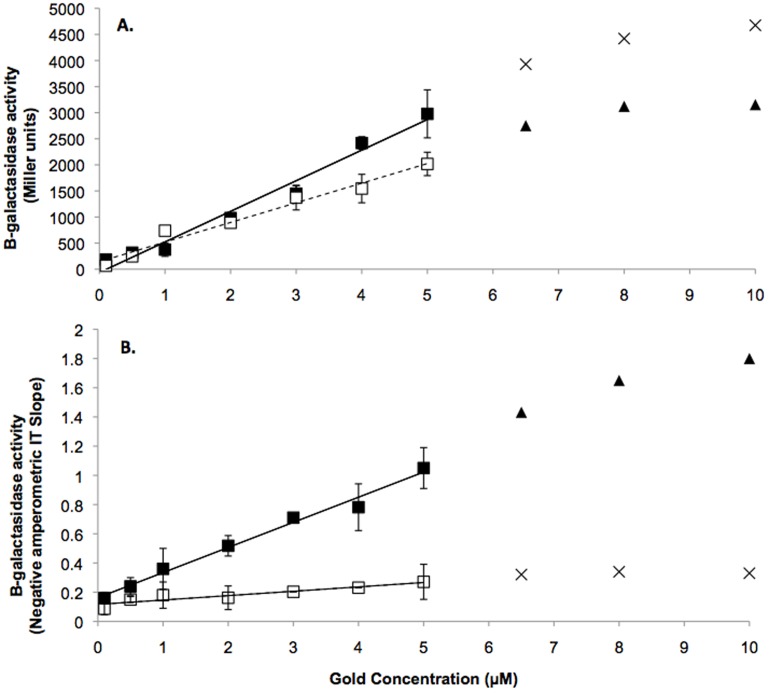
Linear relationship between Au concentrations measurable functional units. These graphs show the concentration of Au measured using the biosensor. (a) Measurements from the β-glactasidase assay, when 0.1 to 5.0 µM of Au(I) and Au(III) were used to induce the biosensor. A linear relationship could be established, outside of this range no relationship between Au concentration and β-glactasidase activity was measured, as shown in triangles for Au(I) and crosses for Au(III). (b) Electrochemistry results when 0.1 to 5.0 µM of Au(I) and Au(III) were used to induce the biosensor and a linear relationship was established, outside of this range no relationship between Au concentration and β-glactasidase activity was measured, as shown in triangles for Au(I) and crosses for Au(III). The solid box and line shows Au(I). The open box and dashed line shows Au(III). The boxes refer to the means, the capped lines show the standard deviation and the line of best fit is also shown (this shows the line formed by the equations used to determine the Au concentration measured from an unknown solution by the biosensor).

The *E. coli* biosensor tested in this study harboured the *golTSB* operon with a transcriptionally fused *lacZ* reporter gene controlled by the GolS regulator (Fig. 1). Using the β-galactosidase assay, a linear relationship was established between MU and Au concentrations with both Au(I) and Au(III) between 0.1 and 5.0 µM (equivalent to 20 to 1000 ng g^−1^ or parts-per-billion (ppb); R^2^ = 0.988 and 0.971 respectively; Fig. 2). Outside of this range (0.01, 0.05 and 6.0 to 10 µM) Au was detected but a greater variation in results was seen and high SD meant that the quantitative results were not statistically significant (Fig. 2).

Gold(I) and Au(III) concentrations can be determined from the β-galactosidase assay using the following equations: [Au(I)] = (MU + 11.292)/590.87 (R^2^ = 0.988) and [Au(III)] = (MU – 134.25)/383.44 (R^2^ of 0.971). At Au concentrations between 0.1 and 1.0 µM the concentration of Au(I) and Au(III) can be determined with the same equation, meaning that a total concentration of Au could be determined ([Au_total_] = [Au(I)] + [Au(III)]). At these concentrations (0.1 to 1 µM) there was no significant difference between the number of MU for Au(I) or (III) (P<0.05). The total amount of Au at these concentrations can be given by [Au_total_] = (MU – 64.04)/482.07 (R^2^ of 0.991).

Results for the electrochemistry were comparable to the β-galactosidase assays. The cells responded to the concentration of Au complexes linearly, although there was a “toxicity” level, in which the cells die because of too much Au in the growth media. This linearity was quantified using chronoamperometry (Fig. 2). Gold concentrations of 0.01 to 10 µM were tested and concentrations of 0.1 to 5 µM could be measured reliably and reproducibly (R^2^ > of 0.95; Fig. 2). Similar to the β-galactosidase assay, the Au(I) species also demonstrated a greater ability to induce the biosensor. The equation generated to determine the concentrations of Au calculated from the of the Amperometric IT Slope (−nAs^−1^) is: [Au(I)] = (−nAs^−1^ - 0.1563)/0.1795 (R^2^ = 0.998) and [Au(III)] = (−nAs^−1^ – 0.094)/0.035 (R^2^ = 0.995).

### Geochemical modeling

**Figure 3:**
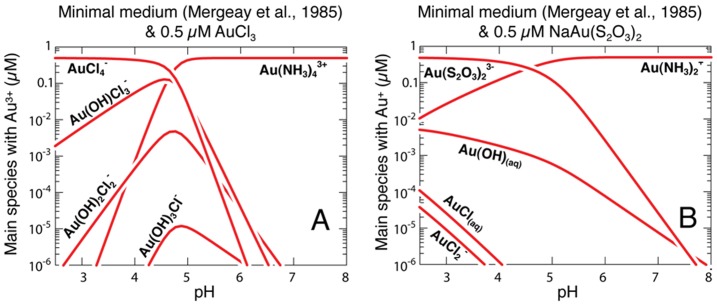
Chemical model showing the speciation of Au in growth media as a function of pH and oxidation state of Au. Chemical model, using Geochemist's Workbench (GWB; [31]), showing the speciation of 0.5 µM NaAu(III)Cl_4(s)_ (a) and 0.5 µM Na_3_Au(I)(S_2_O_3_)_2(s)_ (b), in minimal salt media (MSM, [28]) at pHs 2 to 8.

Using the REACT routine in GWB, titration curves for the addition of up to 10 µM sodium-tetrachloroaurate(III)-hydrate, 10 µM sodium-gold(I)-thiosulfate hydrate, to MSM under thermodynamic equilibrium conditions were calculated (Fig. 3). Results showed that in MSM amended with sodium tetrachloroaurate(III), >99.9 wt.% of Au exists as a Au(III)-tetra-ammonium-complex at pH 7, *i.e.*, the pH of the medium and the selective extractions. In MSM titrated with Au(I)-sodium-thiosulfate >99.9 wt.% of Au exists as a Au(I)-bi-ammonium complex at pH 7. Titration curves at pH 7 with up to 10 µM of sodium-tetrachloroaurate(III)-hydrate and sodium-Au(I)-thiosulfate hydrate, showed that at all concentrations below 10 µM more than >99.9 wt.% of soluble Au consists of Au(III)-tetra-ammonium-complex and Au(I)-bi-ammonium complexes, respectively.

### Transgenic biosensor induction by Au(I)- and Au(III)-complexes in combination with other metal ions

**Figure 4:**
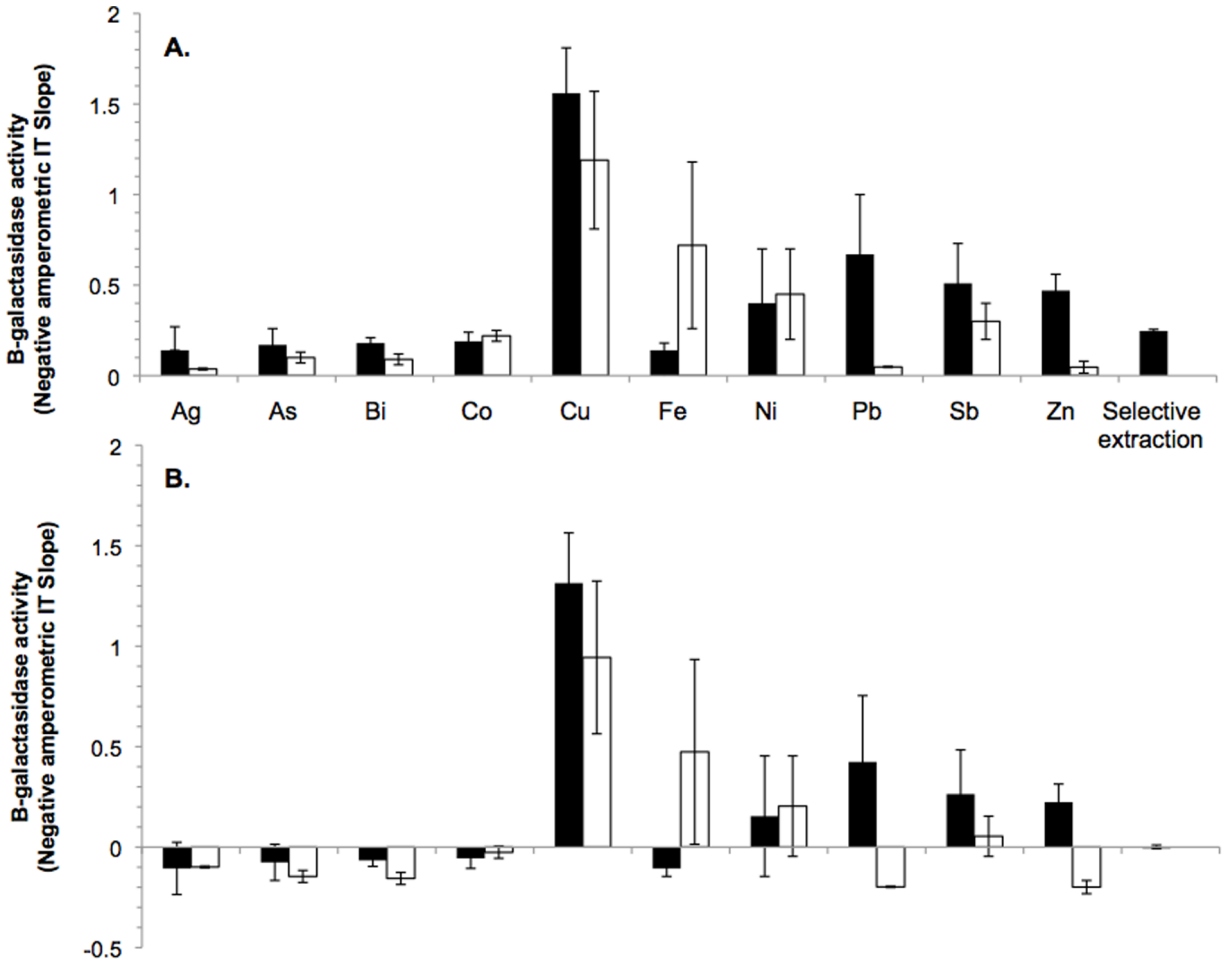
Interference of other metals on the detection and quantification of gold by the biosensor. At the induction stage cross reactivity was tested by adding 5.0 µM Cu(II), Ag(I), Fe(III), Ni(II), Co(II), Zn(II), As(III), Pb(II), Sb(III), or Bi(III) and 0.5 µM Au(I) or Au(III). Measurements were then carried out using an electrochemistry assay as shown in (a). The solid box shows Au(I) and the open box shows Au(III), the capped lines indicate the standard deviation. The calculated β-glactasidase activity would be 0.246 for Au(I) and 0.136 for Au(III), as shown by the line of best fit in Fig. 2. (b) Measurements for the different metals with the expected Au(I) and Au(III) β-glactasidase activity value subtracted.

When testing the electrochemical system with Au(I/III)-complexes in combination with other metals, cross reactivity was observed (Fig. 4). The concentration of the other metals was ten times that of the Au concentration. At 0.5 µM, the −nAs^−1^ for Au(I) averaged 0.24, whereas when calculated using the above equation the −nAs^−1^ would be 0.246. This number was used as the expected −nAs^−1^ for 0.5 µM of Au(I). Similarly for 0.5 µM of Au(III), 0.15 was observed and 0.136 was calculated and used in further analysis. Figure 4b shows the expected −nAs^−1^ for 0.5 µM of Au(I) and Au(III) (*i.e.* 0.246 and 0.136 respectively) subtracted from the measured values. Statistical analysis showed that there were significant differences between the expected −nAs^−1^ and measured −nAs^−1^ across all samples when Au was in the presence of other metals (P >0.1). Cu(II) caused the most cross-reactivity, followed by Fe(III), Ni(II), Pb(II), and Zn(II). Differences were seen in the measurements taken with Au(I) and Au(III). For example, the samples with Au(I) and Pb(II) measured more Au in the sample than was actually present, whereas when Au(III) and Pb(II) were tested the concentration of Au was underestimated (P>0.1). Using the selective extraction, the extracted Au is expected to be in the form Au(I), so the equation for determining the concentration of Au(I) for the electrochemical system was used. The Au which was subjected to selective extraction was measured accurately using the biosensor system (P>0.1).

### Biosensor induction by Au(I)-complexes after selective extractions

**Figure 5:**
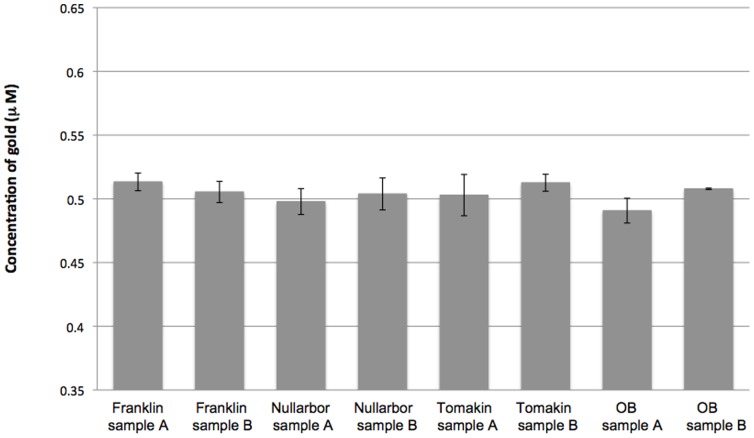
Spiked soil samples subjected to selective extraction and Au quantification. Soil samples from five locations were spiked with Au and then subjected to selective extraction using thiosulfate. The amount of resulting Au was measured with inductively coupled plasma- mass spectrometry (ICP-MS), this was compared to the measurement of Au using the biosensor. The solid boxes shows the concentration of Au as measured using the biosensor when 0.5 µM of Au(I) from the selective extraction was used to induce the biosensor, the capped lines indicated the standard deviation. OB: Ora Banda.

Using the biosensor, concentrations of Au were accurately determined in selective extraction solutions from Au-spiked field soils. The selective extractions had been diluted to contain 0.5 µM of Au based on the initial ICP-MS results. Across the four soils the concentration of Au, as measured using the biosensor, was 0.505 µM (Fig. 5). Using an ANOVA test there was no statistical differences between the measurement of soil extracts using ICP-MS and the biosensor (P>0.1; Fig. 5). There were no apparent differences in the measurement of Au between soil types (P>0.1; Fig. 5).

## Discussion

An in-field, in-line detection method for Au will be beneficial for use in the mining industry, as it will make it possible for exploration geologists to obtain on-site quantification of Au, which is not possible with current technologies. To achieve this, the *gol* genes of *S. enterica* seemingly provide the perfect starting material for the development of a Au biosensor, and our results indicate that this genetic determinant can be used for the quantification of Au ions down to 0.5 µM (20 ppb) and the detection of Au ions down to 0.01 µM (0.4 ppb). Detection of Au using existing technologies in commercial laboratories, such as ICP-MS, allows for the quantification of Au down to 0.025 µM (1 ppb). This puts the biosensor in a similar range of Au detection, making it of great interest to the mining industry. With further refinement, the whole-cell biosensor system may make it possible to quantify Au at levels equal or below that of ICP-MS.

Gold speciation was shown to effect the biosensors ability to measure the concentrations of Au(I) and Au(III). With the colorimetric β-galactosidase assay, the concentrations of Au(I) and Au(III) could be determined using the same equation between 0.1 and 1 µM. Using the electrochemical assay, no linear equation could be derived across the concentrations of Au(I) and Au(III). The induction of *gol* and thus, *lacZ* was more responsive to Au(I) than Au(III), as was seen by the number of MU and the Amperometric IT Slope of the colorimetric and electrochemical assay, respectively. This contradicts the long held belief that Au(III) is more toxic to cells than Au(I) [33], [34], and supports the recent research that shows the toxicity of Au depends on the thermodynamic stability constants of the complexes, as shown for *C. metallidurans* ([18], Nies personal communication). Another possibility for Au(I) having greater potential for inducting the biosensor than Au(III) could be due to differences in the growth of the microorganism. The electrochemical assay does not take into account variations in growth rates and cell numbers, which would affect the amount of the β-galactosidase protein in the cultures. The assumption is made that the cells growing at 0.1 µM of Au(I) or 5.0 µM of Au(III) are progressing along the same growth curve. However, again, these problems are overcome with the use of a selective extraction, as the speciation of Au is controlled by the selective extraction procedure.

A major challenge for the application of biosensors has been the less-than-rigorous testing of the systems under field conditions [16]. As a first step, the whole-cell biosensor was developed in this study and tested against a range of metals (Fig. 4). The metals chosen can be expected to be present along with Au in the soil, hence theoretically interfere with the use of a biosensor [35]. As the electrochemical system would form the basis of a field-based assay, it was chosen to be the system used for testing metal cross-reactivity. It was found that the addition of other metals impaired the ability of the sensor to measure the concentration of Au accurately. This contradicted a previous study by Checa *et al.* (2007) where the induction of *golB* with Cu(II), Ag(I), Zn(II), Cd(II), Hg(II), Fe(II), Co(II), Ni(II), and Pb(II) was investigated. They reported that *gol* was significantly more responsive to Au(III) than to any other metals tested. However, the Checa *et al.* (2007) [25] study varied in a number of aspects from the one presented here. The concentrations of metals tested ranged from 10 µM of Hg(II) to 1 mM of Cu(II) (50 µM of Au was used). These concentrations were not normalized in subsequent analyses. Most importantly, Au(I/III) was mixed with the other metal ions to test if Au can still be detected by the sensor in the present study, whereas the Checa *et al.* (2007) study only investigated one metal at a time.

There may be a number of reasons why the biosensor was unable to accurately detect Au when in the presence of other metals (ratio of Au to metal of 1∶10). Most of these are likely to be due to the inherent limitations of using a whole-cell as a biosensor. A whole-cell biosensor relies upon the induction of genes and the measurement of their gene products: hence, the cells must be incubated with the material that is to be detected. In the case of several metals in the induction solution, their toxicity to cells may inhibit cell growth. Hence, the number of gene products would be lower, resulting in an under-estimation of the concentration of Au in the starting material. This was observed with Ag, As, Bi and Co (Fig. 4). A minimal media was used to grow the cells prior to induction, possibly this media is not optimal for the growth of this microorganism. When elements such as Cu, Fe, and Ni were added to the media, there was possibly an increase in growth rates, hence more β-galactosidase being produced and the concentration of Au would be determined to be higher.

The biosensor can only measure Au as complexes in true solution, so solubilization must be ensured prior to measurement by the biosensor. This was demonstrated by subjecting field samples to a selective extraction technique prior to analysis with the biosensor. Generally, the objective of selective extractions is to determine the amount of an element by using a sequence of reagents with successively stronger binding fractions to extract elements (*e.g.*, [36], [37]). Selective extraction techniques for Au have been used for a long time in geochemical research: to study the association of Au with various soil and other regolith components; to understand how Au is mobilized and trapped in the regolith; to develop effective exploration strategies for Au deposits; and to understand the bioavailability of heavy metals (*e.g.*, [11], [13], [38], [39]).

When Au spiked soils were subjected to selective extraction Au could be effectively recovered. Using the biosensor to measure the concentration of recovered Au from the selective extraction resulted in the accurate quantification of Au, across the range of soils from four different sites (Fig. 5). These results are very promising for the development of a commercially viable biosensor. Improvements in the times taken to extract the Au will be of great benefit to the implementation of a Au biosensor in the mining industry.

Another way of overcoming the intrinsic problems of a cellular biosensor would be to develop a system that does not require the material to be incorporated into the growth media, *i.e.*, no induction phase. This could be achieved via a protein-based system, where proteins, independent of cells, directly interact with Au [40]. Using a protein based sensor may also make it possible to measure far lower concentrations of Au. This has been demonstrated by Bontidean *at al.* [40] who developed a biosensor that could measure femtomolar (10^−15^) concentrations of Cu(II), Cd(II), Hg(II), and Zn(II). However, protein-based biosensors have their own limitations such as low yield of recombinant protein or protein instability with real-life samples. In conclusion, we have presented the ‘proof of concept’ for the development of a cellular Au biosensor. Concentrations of Au ranging from 0.1 to 5 µM (equivalent to 20 to 1000 ng g^−1^ or ppb) were quantified accurately using the biosensor, whilst Au concentrations down to 0.01 µM (0.4 ppb) could be detected.

## Supporting Information

Figure S1 **Map of plasmid created for the biosensor in** ***E.*** ** ***coli.*** The *golTSB* regulon from *Salmonella enterica* serovar typhimurium was integrated into pGEM, then inserted into pAH125 with PstI and EcoRI. This plasmid was then introduced to *E. coli*, this was then used as a gold biosensor.
(TIF)

Table S1 **Geochemistry of soils used for selective extraction.**
(DOCX)
