# Correction: A Whole-Cell Biosensor for the Detection of Gold

**DOI:** 10.1371/annotation/69e42f8e-e942-40eb-a597-7d9ca64552a3

**Published:** 2014-01-22

**Authors:** Carla M. Zammit, Davide Quaranta, Shane Gibson, Anita J. Zaitouna, Christine Ta, Joël Brugger, Rebecca Y. Lai, Gregor Grass, Frank Reith

The authors wish to acknowledge an error in and and correct an error in their use of a reference. The fourth sentence of the seventh paragraph of the Introduction should read:

"A biosensor system, observed by Checa et al. (2011), was capable of detecting Au, quantification of Au was not reported here: However, quantification is crucially important for geochemical exploration [26]."

In addition, the authors wish to acknowledge the relevance of an article 'Cerminati S, Soncini FC, Checa SK. Selective detection of gold using genetically engineered bacterial reporters. Biotechnol Bioeng. 2011 Nov; 108(11):2553-60. doi: 10.1002/bit.23213', which relates to the results described in the present article, as follows:

A Au biosensor system based on the golTSB regulon and a fluorescent reporter protein was previously developed by Cerminati et al. (2011) and dose-response curves were constructed using KAu(CN)2. While Cerminati et al. (2011) suggested that biosensor systems may be useful for quantifying Au in real soil samples this was not tested in their study. Cerminati et al. (2011) developed a Au biosensor system which under "clean" laboratory conditions was capable of detecting environmentally relevant levels of Au. Our study takes further steps towards the development of a field-ready biosensor system with the development and testing of a selective extraction technique for Au, thermodynamic modelling of Au complexes under experimental conditions and the electrochemical testing of the sensor on complex environmental samples.

Similar to the biosensor system presented in Zammit et al. (2013), the Au biosensor developed by Cerminati et al. (2011) was able to quantify Au. However, the gold biosensor developed by Cerminati et al. (2011) itself and the experimental conditions in the Checa et al. (Checa SK, Espariz M, Perez Audero ME, Botta PE, Spinell SV, et al. (2007) Bacterial sensing of and resistance to gold salts. Mol. Mic. 63: 1307-1318.) and Cerminati et al. (2011) studies varied in a number of important aspects from the ones in Zammit et al. (2013). The Cerminati et al. (2011) biosensor was developed using the golTSB genes from S. typhimurium fused to the gene of a green fluorescent protein (GFP) and expressed in E. coli. In contrast, the biosensor developed for this study used the golTSB genes from S. typhimurium transcriptionally fused to a promoterless lacZ reporter cassette, whose activity was measured electrochemically aimed at future in-field-application. These differences may have contributed to the differences in sensitivity of Au detection reported in both studies. The biosensor developed by Cerminati et al. (2011) was able to quantify Au down to 33.23 nM Au(I). The biosensor developed by Zammit et al. (2013) was able to quantify Au down to a detection limit of 10 nM. However, differences may also be a result of the different Au(I/III)-complexes used in both studies to construct standard curves. While KAu(CN)2 was used by Cerminati et al. (2011), Au(I)-thiosulfate and AuHCl4.3H2O were added to the medium by Zammit et al. (2013). The concentrations of metals tested ranged from 10 *M of Hg(II) to 1 mM of Cu(II) (50 *M of Au was used). These concentrations were not normalized in subsequent analyses. Importantly, in Zammit et al. (2013), cross reactivity of Au(I/III) was tested by mixing the complexes mixed with the other metal ions to show that Au can still be detected by the sensor whereas the Checa et al. (2007) and Cerminati et al. (2011) studies only investigated one metal at a time.

