# Supplementary figures and images for: A Whole-Cell Biosensor for the Detection of Gold
Source: PLoS One. 2013 Aug 9;8(8):e69292. doi: 10.1371/journal.pone.0069292 (PMC3739760; doi:10.1371/journal.pone.0069292)

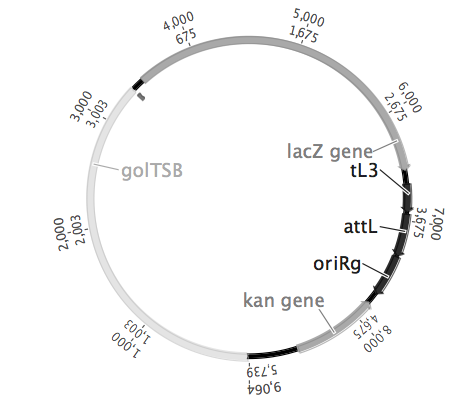

Supplement: Figure S1 — Map of plasmid created for the biosensor in E. coli. The golTSB regulon from Salmonella enterica serovar typhimurium was integrated into pGEM, then inserted into pAH125 with PstI and EcoRI. This plasmid was then introduced to E. coli, this was then used as a gold biosensor. (TIF) [file pone.0069292.s001.tif]
