# Supplementary material for: A Whole-Cell Biosensor for the Detection of Gold
Source: PLoS One. 2013 Aug 9;8(8):e69292. doi: 10.1371/journal.pone.0069292 (PMC3739760; doi:10.1371/journal.pone.0069292)
Supplement: Table S1 — Geochemistry of soils used for selective extraction. (DOCX) [file pone.0069292.s002.docx]

Supplementary Table One.

| **Sample ID** | **dS/m** | **C%** | **Org C%** | **Clay%** | **Silt%** | **Fine sand%** | **Coarse sand%** | **Ca mg/kg** | **K mg/kg** | **Mg mg/kg** | **Na mg/kg** | **S mg/kg** | **Al mg/kg** | **Fe mg/kg** | **Mn mg/kg** |
| --- | --- | --- | --- | --- | --- | --- | --- | --- | --- | --- | --- | --- | --- | --- | --- |
| **Nullabor** | 0.197 | 6.8 | 1.3 | 36.6 | 20.2 | 39.0 | 4.2 | 172000 | 7240 | 11100 | 833 | 682 | 30400 | 18600 | 282 |
| **Ora Banda** | 0.028 | 0.79 | 0.8 | 25.3 | 7.0 | 55.9 | 11.8 | 1400 | 1190 | 1490 | 73.2 | 97 | 54700 | 121000 | 473 |
| **Frankland** | 0.018 | 5.0 | 4.9 | 3.2 | 0.6 | 44.5 | 51.7 | 910 | <20 | 238 | <20 | 230 | 298 | 169 | <10 |
| **Tomakin** | 0.25 | 8.8 | 8.6 | 32.0 | 13.8 | 47.0 | 7.2 | 84.8 | 13100 | 2630 | 334 | 102 | 24600 | 12800 | 99.4 |
